# Supplementary material for: Adverse events related to ultrasound-guided regional anesthesia performed by Emergency Physicians: Systematic review protocol
Source: PLoS One. 2022 Jun 24;17(6):e0269697. doi: 10.1371/journal.pone.0269697 (PMC9231708; doi:10.1371/journal.pone.0269697)
Supplement: S2 File — (DOCX) [file pone.0269697.s002.docx]

**Methods section text:**

The published literature was searched using strategies created by a medical librarian for ultrasound-guided nerve block for emergency department patients. The search strategies were established using a combination of standardized terms and keywords, including but not limited to (ultrasound OR echography) AND (nerve block OR regional anesthesia OR conduction anesthesia) AND (emergency department OR acute care OR emergency physician OR emergency medical services OR emergency ward OR trauma center). The search was run in April 2021 without any filters in the databases Ovid Medline 1946-, Embase.com 1947-, Scopus 1823-, Cochrane Central Register of Controlled Trials, PubMed, and Clinicaltrials.gov. A total of 1,404 citations were exported to Endnote. 634 duplicates were assumed to be accurately identified and removed for a total of 770 unique citations. Full electronic search strategies are provided in the supplementary material.

The literature search was executed again on March 9, 2022. 220 new citations were found. 89 duplicates were removed for a total of 131 unique citations added to the pool of citation results.

**Supplement:**

Total: 1624

Duplicates: 723
Unique: 901

Ovid Medline: 232
Embase: 715
Scopus: 448
Cochrane Central: 120
PubMed (using “not Medline” filter): 59
Clinicaltrials.gov: 50

**Ovid Medline
208 results on 4/22/21**

**24 results on 3/09/22 with limit 4 to dt=20210422-20220309**

Ovid MEDLINE(R) ALL <1946 to April 21, 2021>

1 exp Emergency Medical Services/ or exp hospital emergency service/ or emergicenter*.mp. or (acute adj2 (setting or care or ward or hospital)).mp. or ((Emergency or emergencies) adj3 (care or service* or dispatch* or department* or unit* or ward* or room* or center* or centre* or system* or stay* or admit* or admission* or evaluation* or assess* or medicine or Nurs* or physician* or patient* or clinician or personnel or accidents)).mp. or ((EMS or ED or ER) adj3 (care or service* or dispatch* or department* or unit* or ward* or room* or center* or centre* or system* or stay* or admit* or admission* or evaluation* or assess* or Nurs* or physician* or patient* or personnel)).mp. or (("a&e" or "a & e") adj2 (department* or admission* or admitted)).mp. or (trauma adj1 (center* or centre* or department*)).mp. 321901

2 exp Nerve Block/ or exp Conduction Anesthesia/ or (nerve adj2 (block or blocks or blockade or blockades or blocking)).mp. or ((conduction or regional or epidural or peridural or extradural or caudal or local or infiltration or spinal) adj2 (anesthesia* or anaesthesia*)).mp. or (huneke adj1 neural-therapy).mp. or (Chemical adj1 (Neurolysis or Neurolyses)).mp. or Chemodenervation*.mp. or ((Brachial-Plexus or brachial or cervical-plexus or cervical or intercostal-nerve or lumbar-plexus or transversus-abdominus-plane or retroocular) adj1 (block or blocks or blockade* or anesthesia or anaesthesia)).mp. or ((conduction or autonomic or neurogenic) adj1 (block or blocks or blocker or blockade)).mp. 100167

3 Ultrasonography/ or exp doppler ultrasonography/ or exp interventional ultrasonography/ or exp Elasticity Imaging Techniques/ or exp Focused Assessment with Sonography for Trauma/ or exp Endosonography/ or Ultrasound*.mp. or ultrasonic*.mp. or doptone.mp. or echography.mp. or echogram.mp. or echographic.mp. or echoscopy.mp. or echosound.mp. 497500

4 1 and 2 and 3 208

**Embase.com**

**627 results on 4/22/21
88 results on 3/09/22 with date limit #4 AND [22-04-2021]/sd NOT [10-03-2022]/sd**

**1.** 'echography'/exp OR Ultrasound*:ti,ab,kw,de OR ultrasonic*:ti,ab,kw,de OR doptone:ti,ab,kw,de OR echography:ti,ab,kw,de oR echogram:ti,ab,kw,de OR echographic:ti,ab,kw,de oR echoscopy:ti,ab,kw,de OR echosound:ti,ab,kw,de OR echography:ti,ab,kw,de = 1,173,121

2. ‘nerve block’/de OR ‘regional anesthesia’/de OR ‘intravenous regional anesthesia’/de OR ‘brachial plexus anesthesia’/exp OR ‘cervical plexus block’/exp OR ‘intercostal nerve block’/exp OR ‘lumbar plexus block’/exp OR ‘transversus abdominis plane block’/exp OR (nerve near/2 (block OR blocks OR blockade OR blockades OR blocking)):ti,ab,kw,de OR ((conduction OR regional OR epidural OR peridural OR extradural OR caudal OR local OR infiltration OR spinal) near/2 (anesthesia* OR anaesthesia*)):ti,ab,kw,de OR (huneke near/1 (neural-therapy)):ti,ab,kw,de OR (Chemical near/1 (Neurolysis OR Neurolyses)):ti,ab,kw,de OR Chemodenervation*:ti,ab,kw,de OR ((Brachial-Plexus OR brachial OR cervical-plexus OR cervical OR intercostal-nerve OR lumbar-plexus OR transversus-abdominus-plane OR retroocular) near/1 (block or blocks Or blockade* OR anesthesia OR anaesthesia)):ti,ab,kw,de OR ((conduction OR autonomic OR neurogenic) near/1 (block OR blocks OR blocker OR blockade)):ti,ab,kw,de = 159,273

**3.** ‘emergency health service’/exp OR ‘emergency ward’/exp OR emergicenter*:ti,ab,kw,de,kw,de OR (acute NEAR/2 (setting OR care OR ward OR hospital)):ti,ab,kw,de OR ((trauma NEAR/1 (center* OR centre* OR department*)):ti,ab,kw,de) OR ((Emergency OR emergencies) near/3 (care OR service* OR dispatch* OR department* OR unit* OR ward* OR room* OR center* OR centre* OR system* OR stay* or admit* or admission* or evaluation* OR assess* OR medicine OR Nurs* OR physician* OR clinician* OR personnel OR patient* OR accidents)):ti,ab,kw,de,kw,de OR ((EMS OR ED OR ER) near/3 (care OR service* OR dispatch* OR department* OR unit* OR ward* OR room* OR center* OR centre* OR system* OR stay* or admit* or admission* or evaluation* OR assess* OR Nurs* OR physician* OR patient* OR personnel)):ti,ab,kw,de,kw,de OR (('a&e' OR 'a & e') NEAR/2 (department* OR admission* OR admitted)):ti,ab,kw,de = 497,813

4. 1 AND 2 AND 3 = 627

**Scopus**

**381 results on 4/22/21
67 results on 3/09/22 with date limit #4 AND ( LIMIT-TO ( PUBYEAR , 2022 ) OR LIMIT-TO ( PUBYEAR , 2021 ) )**

**1.** **(TITLE-ABS-KEY (**Ultrasound* OR ultrasonic* OR doptone OR echography oR echogram OR echographic oR echoscopy OR echosound OR echography))

2. **(TITLE-ABS-KEY** (nerve w/2 (block OR blocks OR blockade OR blockades OR blocking))) OR **(TITLE-ABS-KEY** ((conduction OR regional OR epidural OR peridural OR extradural OR caudal OR local OR infiltration OR spinal) w/2 (anesthesia* OR anaesthesia*))) OR **(TITLE-ABS-KEY** (huneke w/1 (neural-therapy))) OR **(TITLE-ABS-KEY** (Chemical w/1 (Neurolysis OR Neurolyses))) OR **(TITLE-ABS-KEY (**Chemodenervation*)) OR **(TITLE-ABS-KEY** ((Brachial-Plexus OR brachial OR cervical-plexus OR cervical OR intercostal-nerve OR lumbar-plexus OR transversus-abdominus-plane OR retroocular) w/1 (block or blocks Or blockade* OR anesthesia OR anaesthesia))) OR **(TITLE-ABS-KEY** ((conduction OR autonomic OR neurogenic) w/1 (block OR blocks OR blocker OR blockade)))

**3.** **(TITLE-ABS-KEY (**emergicenter*)) OR **(TITLE-ABS-KEY** (acute W/2 (setting OR care OR ward OR hospital))) OR **(TITLE-ABS-KEY** ((trauma W/1 (center* OR centre* OR department*))) ) OR **(TITLE-ABS-KEY** ((Emergency OR emergencies) w/3 (care OR service* OR dispatch* OR department* OR unit* OR ward* OR room* OR center* OR centre* OR system* OR stay* or admit* or admission* or evaluation* OR assess* OR medicine OR Nurs* OR physician* OR clinician* OR personnel OR patient* OR accidents))) OR **(TITLE-ABS-KEY** ((EMS OR ED OR ER) w/3 (care OR service* OR dispatch* OR department* OR unit* OR ward* OR room* OR center* OR centre* OR system* OR stay* or admit* or admission* or evaluation* OR assess* OR Nurs* OR physician* OR patient* OR personnel)))

4. 1 AND 2 AND 3 = 627

**PubMed Central**

**48 results using NOT MEDLINE filter
11 results on 3/09/22 with date filter (2021/4/22:2022/3/9[pdat])**

((“Nerve Block”[mesh] OR “Conduction Anesthesia”[mesh] OR “nerve block”[tiab] OR “nerve blocks”[tiab] OR “nerve blocking”[tiab] OR “nerve blockade”[tiab] OR “conduction anesthesia”[tiab] OR “regional anesthesia”[tiab] OR “conduction anaesthesia”[tiab] OR “regional anaesthesia”[tiab] OR “chemical neurolysis”[tiab] OR chemodenervation[tiab] OR “brachial plexus block”[tiab] OR “cervical plexus block”[tiab] OR “brachial block”[tiab] OR “lumbar plexus block”[tiab] OR “transversus abdominus plane block”[tiab] OR “retrocolular block”[tiab])

AND

("Emergency Service, Hospital"[Mesh] OR "Emergency Medical Services"[mesh] OR emergicenter*[tiab] OR "acute care"[tiab] OR "acute hospital"[tiab] OR "trauma center"[tiab] OR "trauma department"[tiab] OR "trauma center" [tiab] OR "trauma centre"[tiab] OR "trauma unit"[tiab] OR "emergency Care"[tiab] OR "emergency service"[tiab] OR "emergency department"[tiab] OR "emergency departments"[tiab] OR "emergency center"[tiab] OR "emergency centre"[tiab] OR "emergency ward"[tiab] OR "emergency wards"[tiab] OR "emergency medicine"[tiab] OR "ED care"[tiab] OR "emergency room"[tiab] OR "EMS care"[tiab])

AND
(Ultrasonography[mesh] or Ultrasound*[tiab] or ultrasonic*[tiab] or doptone[tiab] or echography[tiab] or echogram[tiab] or echographic[tiab] or echoscopy[tiab] or echosound[tiab]))

NOT (medline[Filter])

**Cochrane Central**

**105 results on 4/22/21
15 results on 3/09/22 with custom date range 22/04/2021 to 09/03/2022**

**1.** Ultrasound*:ti,ab,kw OR ultrasonic*:ti,ab,kw OR doptone:ti,ab,kw OR echography:ti,ab,kw oR echogram:ti,ab,kw OR echographic:ti,ab,kw oR echoscopy:ti,ab,kw OR echosound:ti,ab,kw OR echography:ti,ab,kw

2. (nerve near/2 (block OR blocks OR blockade OR blockades OR blocking)):ti,ab,kw OR ((conduction OR regional OR epidural OR peridural OR extradural OR caudal OR local OR infiltration OR spinal) near/2 (anesthesia* OR anaesthesia*)):ti,ab,kw OR (huneke near/1 (neural-therapy)):ti,ab,kw OR (Chemical near/1 (Neurolysis OR Neurolyses)):ti,ab,kw OR Chemodenervation*:ti,ab,kw OR ((Brachial-Plexus OR brachial OR cervical-plexus OR cervical OR intercostal-nerve OR lumbar-plexus OR transversus-abdominus-plane OR retroocular) near/1 (block or blocks Or blockade* OR anesthesia OR anaesthesia)):ti,ab,kw OR ((conduction OR autonomic OR neurogenic) near/1 (block OR blocks OR blocker OR blockade)):ti,ab,kw

**3.** emergicenter*:ti,ab,kw OR (acute NEAR/2 (setting OR care OR ward OR hospital)):ti,ab,kw OR ((trauma NEAR/1 (center* OR centre* OR department*)):ti,ab,kw) OR ((Emergency OR emergencies) near/3 (care OR service* OR dispatch* OR department* OR unit* OR ward* OR room* OR center* OR centre* OR system* OR stay* or admit* or admission* or evaluation* OR assess* OR medicine OR Nurs* OR physician* OR clinician* OR personnel OR patient* OR accidents)):ti,ab,kw OR ((EMS OR ED OR ER) near/3 (care OR service* OR dispatch* OR department* OR unit* OR ward* OR room* OR center* OR centre* OR system* OR stay* or admit* or admission* or evaluation* OR assess* OR Nurs* OR physician* OR patient* OR personnel)):ti,ab,kw

4. 1 AND 2 AND 3 = 627

**Clinicaltrials.gov**

**35 results on 4/22/21
15 results on 3/09/22 with date limt “Last update posted from 04/22/2021 to 03/09/2022”**

(echography-guided OR ultrasound-guided) AND (nerve block) AND ("emergency medical services" OR "emergency department" OR "emergency departments" OR "emergency room")
