# Supplementary material for: Adverse events related to ultrasound-guided regional anesthesia performed by Emergency Physicians: Systematic review protocol
Source: PLoS One. 2022 Jun 24;17(6):e0269697. doi: 10.1371/journal.pone.0269697 (PMC9231708; doi:10.1371/journal.pone.0269697)
Supplement: Attachment — Submitted filename: Response to reviewers - 3.30.22.docx [file pone.0269697.s004.docx]

Dear Dr. Bahl and PLoS One Reviewers,

Thank you for your insightful comments on our submission and for your recommendations on improving our work. Overall, we felt your suggestions were helpful and improve the quality of our work. We also appreciate you highlighting some of the formatting particulars that were initially overlooked.

Below, have included our responses in bold beside the original comments, indicating how we have addressed the concerns in our revised manuscript.

Additionally, we noticed that one of the authors names (Dr. Lee) had a misspelling in his name on the title page. This has been corrected.

Sean Stickles, MD (corresponding author)

----

Journal Requirements:

When submitting your revision, we need you to address these additional requirements.

1. Please ensure that your manuscript meets PLOS ONE's style requirements, including those for file naming. The PLOS ONE style templates can be found at 
<https://journals.plos.org/plosone/s/file?id=wjVg/PLOSOne_formatting_sample_main_body.pdf> and 
<https://journals.plos.org/plosone/s/file?id=ba62/PLOSOne_formatting_sample_title_authors_affiliations.pdf> -- **This has been corrected.**

2. Please ensure that you include a title page within your main document. We do appreciate that you have a title page document uploaded as a separate file, however, as per our author guidelines (<http://journals.plos.org/plosone/s/submission-guidelines#loc-title-page>) we do require this to be part of the manuscript file itself and not uploaded separately.

Could you therefore please include the title page into the beginning of your manuscript file itself, listing all authors and affiliations.

**--Thank you for pointing this out, this has been added to the manuscript file itself.**

3. We note that you have indicated that data from this study are available upon request. PLOS only allows data to be available upon request if there are legal or ethical restrictions on sharing data publicly. For information on unacceptable data access restrictions, please see <http://journals.plos.org/plosone/s/data-availability#loc-unacceptable-data-access-restrictions>.

In your revised cover letter, please address the following prompts:

a) If there are ethical or legal restrictions on sharing a de-identified data set, please explain them in detail (e.g., data contain potentially identifying or sensitive patient information) and who has imposed them (e.g., an ethics committee). Please also provide contact information for a data access committee, ethics committee, or other institutional body to which data requests may be sent.

b) If there are no restrictions, please upload the minimal anonymized data set necessary to replicate your study findings as either Supporting Information files or to a stable, public repository and provide us with the relevant URLs, DOIs, or accession numbers. Please see <http://www.bmj.com/content/340/bmj.c181.long> for guidelines on how to de-identify and prepare clinical data for publication. For a list of acceptable repositories, please see <http://journals.plos.org/plosone/s/data-availability#loc-recommended-repositories>.

**--As a protocol paper, we do not have any current data sets to present. However, we have provided clarification that our intent is to publish all available collected data extracted from our review.**

We will update your Data Availability statement on your behalf to reflect the information you provide

Please review your reference list to ensure that it is complete and correct. If you have cited papers that have been retracted, please include the rationale for doing so in the manuscript text, or remove these references and replace them with relevant current references. Any changes to the reference list should be mentioned in the rebuttal letter that accompanies your revised manuscript. If you need to cite a retracted article, indicate the article’s retracted status in the References list and also include a citation and full reference for the retraction notice.
**--All references listed are correct at this time to the best of our knowledge.**

[Note: HTML markup is below. Please do not edit.]

Reviewers' comments:

Reviewer's Responses to Questions

**Comments to the Author**

1. Does the manuscript provide a valid rationale for the proposed study, with clearly identified and justified research questions?

The research question outlined is expected to address a valid academic problem or topic and contribute to the base of knowledge in the field.

Reviewer #1: Yes

Reviewer #2: Yes

2. Is the protocol technically sound and planned in a manner that will lead to a meaningful outcome and allow testing the stated hypotheses?

The manuscript should describe the methods in sufficient detail to prevent undisclosed flexibility in the experimental procedure or analysis pipeline, including sufficient outcome-neutral conditions (e.g. necessary controls, absence of floor or ceiling effects) to test the proposed hypotheses and a statistical power analysis where applicable. As there may be aspects of the methodology and analysis which can only be refined once the work is undertaken, authors should outline potential assumptions and explicitly describe what aspects of the proposed analyses, if any, are exploratory.

Reviewer #1: Yes

Reviewer #2: Yes

3. Is the methodology feasible and described in sufficient detail to allow the work to be replicable?


Descriptions of methods and materials in the protocol should be reported in sufficient detail for another researcher to reproduce all experiments and analyses. The protocol should describe the appropriate controls, sample size calculations, and replication needed to ensure that the data are robust and reproducible.

Reviewer #1: Yes

Reviewer #2: Yes

4. Have the authors described where all data underlying the findings will be made available when the study is complete?

The [PLOS Data policy](https://journals.plos.org/plosone/s/materials-and-software-sharing) requires authors to make all data underlying the findings described in their manuscript fully available without restriction, with rare exception, at the time of publication. The data should be provided as part of the manuscript or its supporting information, or deposited to a public repository. For example, in addition to summary statistics, the data points behind means, medians and variance measures should be available. If there are restrictions on publicly sharing data—e.g. participant privacy or use of data from a third party—those must be specified.

Reviewer #1: No

Reviewer #2: Yes

5. Is the manuscript presented in an intelligible fashion and written in standard English?

PLOS ONE does not copyedit accepted manuscripts, so the language in submitted articles must be clear, correct, and unambiguous. Any typographical or grammatical errors should be corrected at revision, so please note any specific errors here.

Reviewer #1: Yes

Reviewer #2: Yes

6. Review Comments to the Author

Please use the space provided to explain your answers to the questions above and, if applicable, provide comments about issues authors must address before this protocol can be accepted for publication. You may also include additional comments for the author, including concerns about research or publication ethics.

You may also provide optional suggestions and comments to authors that they might find helpful in planning their study.


(Please upload your review as an attachment if it exceeds 20,000 characters)

Reviewer #1: The authors present a protocol for a systematic review of existing literatures published on the safety and variety of ultrasound guided nerve blocks performed by emergency physicians. While there are several systematic reviews looking at regional nerve blocks as well as ultrasound guided regional nerve blocks, I would agree with the authors that none have been published looking at the specific topic which they propose here. Their protocol is precise, well written, and feasible. I commend the authors for their efforts and am excited to see this systematic review published.

There are several items that should be addressed prior to publication of this protocol:
1. In the methods section clarity is needed regarding if studies performed outside of the ED will be considered. The interventions section currently states that all ultrasound guided studies will be considered. However, the remainder of your protocol seems to suggest that only ED based studies will be included. **– This was clarified.**
2. According to PRISMA guidelines for systematic review protocol, your section discussing search strategy should include a complete draft search strategy for at least one database including planned limits such that it could be repeated. – **This is now included.**
3. Should consider adding funding source as a collected data item from the studies. –**This is now included.**

4. If intended by the authors to allow for open access to their data, there is currently no description of how data will be made available after publication. –**We anticipate all data collected will be included in a table or tables in the final manuscript.** **We have added a sentence to the manuscript indicating such.**
5. Consider including your standardized data collection form as a supplementary figure.—**This is now included.**

Reviewer #2: I believe that this systematic review will provide useful information at a time when emergency provider performed USGRA is rapidly increasing. There is a lot of information regarding safety and efficacy of these procedures in orthopedic and anesthesiology literature, but this same body of research does not exist in emergency medicine.

Eligibility criteria - it may be relevant to include a subgroup analysis of geriatric patients (>65yo), as this is a patient population that oftentimes benefits greatly from USGRA. There is an especially large body of orthopedic literature stating that fascia iliaca compartment blocks/PENG blocks, femoral nerve blocks, etc.) decrease need for breakthrough pain/opioid medications, decrease hospital length of stay and morbidity/mortality, decrease time to post-surgical ambulation, decreased PNA/UTIs/nosocomial infections after surgery. The population that sustains these hip fractures are often >65yo, which may warrant further analysis.—**This is now included.**

Lines 29-32 state that "USGRA is not without complications, which can include procedural pain, infection, nerve injury, blood vessel injury, allergic reactions, and the potentially fatal local anesthetic systemic toxicity, among other adverse events." There is a lack of substantial evidence regarding USGRA effects on detecting compartment syndrome. This may specifically be an adverse event/sequelae of extremity injuries that is worth investigating as this is oftentimes a concern that orthopedics has regarding administration of these nerve blocks.—**This is now included.**

7. PLOS authors have the option to publish the peer review history of their article ([what does this mean?](https://journals.plos.org/plosone/s/editorial-and-peer-review-process#loc-peer-review-history)). If published, this will include your full peer review and any attached files.


If you choose “no”, your identity will remain anonymous but your review may still be made public.


**Do you want your identity to be public for this peer review?** For information about this choice, including consent withdrawal, please see our [Privacy Policy](https://www.plos.org/privacy-policy).

Reviewer #1: No

Reviewer #2: No
